# Disease Profiling for Computerized Peer Support of Ménière's Disease

**DOI:** 10.2196/rehab.4109

**Published:** 2015-09-03

**Authors:** Jyrki Rasku, Ilmari Pyykkö, Hilla Levo, Erna Kentala, Vinaya Manchaiah

**Affiliations:** ^1^School of Information SciencesTampere UniversityTampereFinland; ^2^Hearing and Balance Research UnitDepartment of OtorhinolaryngologyTampere UniversityTampereFinland; ^3^Department of OtolaryngologyUniversity of HelsinkiHelsinkiFinland; ^4^Department of Speech and Hearing SciencesLamar UniversityBeaumont, TXUnited States; ^5^The Swedish Institute for Disability ResearchDepartment of Behavioral Sciences and LearningLinköping UniversityLinköpingSweden; ^6^Audiology IndiaMysoreIndia

**Keywords:** Ménière’s disease, diagnosis, disease profiling, peer-support, participation restriction, activity limitation, enablement, machine learning

## Abstract

**Background:**

Peer support is an emerging form of person-driven active health care. Chronic conditions such as Ménière’s disease (a disorder of the inner ear) need continuing rehabilitation and support that is beyond the scope of routine clinical medical practice. Hence, peer-support programs can be helpful in supplementing some of the rehabilitation aspects.

**Objective:**

The aim of this study was to design a computerized data collection system for the peer support of Menière’s disease that is capable in profiling the subject for diagnosis and in assisting with problem solving.

**Methods:**

The expert program comprises several data entries focusing on symptoms, activity limitations, participation restrictions, quality of life, attitude and personality trait, and an evaluation of disease-specific impact. Data was collected from 740 members of the Finnish Ménière’s Federation and utilized in the construction and evaluation of the program.

**Results:**

The program verifies the diagnosis of a person by using an expert system, and the inference engine selects 50 cases with matched symptom severity by using a nearest neighbor algorithm. These cases are then used as a reference group to compare with the person’s attitude, sense of coherence, and anxiety. The program provides feedback for the person and uses this information to guide the person through the problem-solving process.

**Conclusions:**

This computer-based peer-support program is the first example of an advanced computer-oriented approach using artificial intelligence, both in the profiling of the disease and in profiling the person’s complaints for hearing loss, tinnitus, and vertigo.

## Introduction

Ménière’s disease is a disorder of the inner ear that causes spontaneous episodes of vertigo, fluctuating hearing loss, tinnitus, and fullness of the ear [1]. The impact of this complex chronic condition varies greatly from person to person and from time to time, depending on the vertigo attacks experienced [2]. However, generally this condition can result in physical, mental, and social consequences to both the person affected and their significant others [3], causing a poorer health-related quality of life [4]. Even though people with Ménière’s disease are subjected to extensive clinical examination (eg, medical, audiological, and psychosocial evaluations), the diagnosis of this condition is often symptom-based.

There are medical therapies available for this condition (eg, medication, surgery). However, there is no permanent cure from medical therapy, and often there is a requirement for lifestyle changes. Hence, there is a great need for an appropriate rehabilitation program for people with the Ménière’s disease and their significant others to help them learn various coping strategies to live well with this condition.

Increasing the knowledge of the impact of the disease (eg, life consequences in terms of activity limitations and participation restrictions) may be an important first step in rehabilitation. This may assist in attitude change, the acceptance of the condition, and lifestyle modification. Moreover, when dealing with chronic conditions, it has been recognized that “social participation,” in addition to clinical and medical management, may bring many advantages in terms of improving the adherence and compliance to therapy, as well as improving coping with the condition, thereby resulting in better health outcomes. For example, a recent study in multiple sclerosis suggested that social participation was significantly associated with health-related quality of life [5]. Hence, we suggest that social participation should be an important intervention component of chronic conditions such as Ménière’s disease.

Peer support is a salient concept in health care, which considers the patient’s social relationship and social participation as important components. Peer support is defined as the “system of giving and receiving help founded on key principles of respect, shared responsibility, and mutual agreement of what is helpful” [6]. Moreover, “peer support has been defined by the fact that people who have like experiences can better relate and can consequently offer more authentic empathy and validation” [7]. Various forms of peer support exist in health care (ie, person-to-person support, telephone calls, and Internet-based support), and these have become more important in recent years as medical developments and medical technology have provided the increasing challenges of disease with alternative treatment possibilities [8-10]. Furthermore, the outcome of modern health care is increasingly focused on the improvement of the quality of life, and people’s expectations have subsequently increased [11]. Therefore, the expectations of treatment have been that the therapy will lead to a complete healing of the conditions and will provide a normal state of health [12]. However, complete healing of the condition seldom occurs in chronic diseases like Ménière’s disease. The modern approach to improvement emphasizes that in the life of all human beings, social participation is vital. This goal-directed behavior is one of the core constructs. Needless to say, medical therapy seldom achieves these goals whereas peer support commonly focuses on such items. Thus, in peer support the regaining of function is an active process, in that the person should work toward solving their problems.

Peer-support programs give patients an opportunity to actively participate in their own rehabilitation. However, it is important to recognize that peer support is important in all levels of health care delivery, not just in rehabilitation [13]. Person-to-person peer support is based on voluntary activity and is usually constrained by the limited numbers of volunteers. Recently, computerized peer-support programs have been introduced, especially in behavioral disorders [14]. So far, for inner-ear diseases there are a limited number of Internet-based peer-support systems available. One such example is a system developed in Sweden that is aimed at the alleviation of tinnitus and comprises 6 sessions [15]. Most of these sessions entail teaching relaxation techniques and how to avoid the adverse effects of tinnitus with sound therapy. The computer has several advantages: it is anonymous for the supported person, is indefatigable, and can serve hundreds—and even thousands—of people at the same time. Guidance can be tailored to the level of best expertise. And the program can be repeated, restarted, and continued as needed. However, these programs are usually Internet-based implementations, and the interface lacks the capability to profile the person’s complaints and personal needs. Nevertheless, the tinnitus program and programs developed for behavioral disorders demonstrate that a computer-based peer-support system is both realistic and acceptable and provides results as good as a form of person-to-person peer-support [14,15].

## Methods

### Overview

Disease profiling is the first important step in dealing with any chronic condition. Disease profiling should not only consist of medical diagnosis, but should also focus on understanding the impact of the condition on wider life activities. This information is important in planning the management of the disease, especially in tailoring the rehabilitation to meet the needs of the specific individuals. In conditions such as Ménière’s disease where it is a common practice to diagnose based on symptoms, an advanced computer-based system can be applied in disease profiling. Such an approach can be extremely beneficial in self-directed Internet-based and/or computer-based peer-support programs.

The aim of the present study is to develop an intelligent computer-based peer-support system that is capable of assessing Ménière’s disease and profiling the impact of the condition. By applying artificial intelligence and knowledge-based systems, computerized peer-support can be tailored to meet individual needs. The program collects data on the participant’s Ménière’s disease symptoms, activity limitations, participation restrictions, personality traits, moods, and attitudes. It then classifies these in terms of the problems experienced from the disease on a personal level using the International Classification of Functioning, Disability, and Health (ICF) [16]. The program runs interactively and the patient’s profile ultimately controls the program flow so that the computerized peer-support is tailored for individual needs.

### Data Collection

Permission was obtained from the Finnish Ménière’s Federation (FMF; Suomen Meniere-Liitto Ry) to contact their members, asking them to complete an extensive questionnaire on symptoms related to Ménière’s disease. Under Finnish law, this kind of survey study conducted within a patient support organization does not need ethical approval. The sample comprised 1200 individuals. They were sent a 26-page questionnaire by mail, together with a stamped addressed envelope for the return of their responses. These questionnaires have previously been used in our studies on Ménière’s disease [17-19]. A total of 781 candidates returned the questionnaire (65% response rate). Of these, 571 were female and 210 were male, and 740 of the questionnaires were adequately completed. The mean age of respondents was 62.3 years (SD 11.5 years), and their Ménière’s disease-specific symptoms had lasted an average of 16.2 years (SD 11.2 years).

All of the questionnaires were administered in Finnish. The questionnaires comprised both disease-specific and impact-related questions. An otoneurology questionnaire [20] with 86 questions was used to evaluate the characteristics of the otologic disease. A EuroQol EQ-5D tool was used to study the general health-related quality of life [21]. The EuroQol EQ-5D tool has a test-retest reliability of 0.66 and has validity in relation to other generic health-related quality of life measures such as the 36-item Short Form Health Survey (SF-36) [22]. A short version of the sense of coherence (SOC) questionnaire [23] was used to evaluate personality traits. The short SOC scale internal consistency ranges from 0.74 to 0.91, and has a test-retest reliability of 0.54 [23]. A short form of the post-traumatic growth inventory (PTGI-SF) [24] was used to assess improvements caused by the program. The PTGI-SF has a reliability of 0.90 and is very close to the reliability of the full version of the PTGI [24]. Activity limitations were evaluated using the international tinnitus inventory (ITI) [25] with 8 questions, and the hearing disability and handicap scale (HDHS) [26] with 10 questions. The internal consistency of the ITI ranged from 0.87 to 0.91 [25] and HDHS ranged from 0.81 to 0.89 [27]. Sound localization was evaluated using 4 questions based on the hearing measurement scale (HMS) [28,29]. Dizziness and vertigo were evaluated using the 8 questions about vertigo and dizziness [30,31], with an internal consistency ranging from 0.75 to 0.82. Participation restrictions were evaluated using the Participation Restriction Scale [32], which consisted of 30 questions. Disease-specific impact assessment was done using a mixture of open-ended and closed questions. The questionnaire based on the ICF was used to classify the impact of the disease at the individual level [18]. Anxiety or nervousness was evaluated using a 15D questionnaire, which measures general health-related quality of life, by asking the subject: “Does vertigo, hearing loss, or tinnitus cause you anxiety or nervousness?” [33]. A specific question on vitality was also based on a question presented in the 15D [33]. The 15D scale has been found to have appropriate content and construct validity. The repeatability coefficients of the scale are 92%-100% depending on the dimension, and a high sensitivity is achieved as demonstrated by its discriminatory power [34].

Zaphiris et al have estimated the ideal amount of information to be included in Web pages targeted toward older people [35]. We have followed these guidelines in our program.

### Disease Profiling

The complaint history was inputted into the inference engine to assess the diagnosis. In this process, the program uses 2 classes of information: (1) necessary and (2) supportive data. The class of signs depends on the disease so that one sign can be necessary and other supportive. A necessary sign is indicative for Ménière’s disease. Supportive signs suggest the condition, but their presence is not obligatory. The program uses a pattern recognition algorithm in classifying the diseases causing vertigo, which has been described in detail elsewhere [36,37]. Based on the outcome of the inference engine, different probability scores are provided. One probability score indicates the outcome when all necessary questions have been answered, and the other when there are missing data resulting in uncertainty. In calculation of the probability values, the person data is compared with the optimal data.

**Figure 1:**
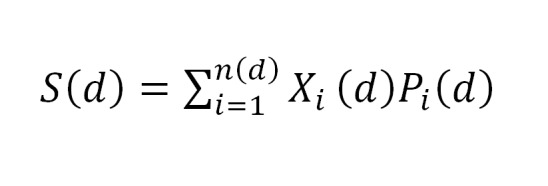
Disease scoring formula.

The knowledge base contains extensive descriptors of 14 diseases causing vertigo. In the program, a descriptor defines the mapping between a disease and answers to the questionnaire’s symptoms and to medical history questions. For a disease *d* there are *n(d)* questions, and a score *S(d)* for the disease as defined below (Figure 1).

In the formula above, the binary variable *X*_*i*_ *(d)* has the value of 1 if a user has provided an answer *i* concerning the disease *d* and otherwise zero. Variable *P*_*i*_ *(d)* is the significance of the question *i* for disease *d*. By this reasoning, we select the diagnosis of disease *d* where the score *S(d)* is the highest. A detailed description of disease scoring as well as handling missing data and dealing with the uncertainty of reasoning has been presented in a previous report [38].

### System Architecture

The program’s user interface was designed to be simple due to the age group of the potential users. The program presents questions and statements to the user in a thematic series. The user generally sorts text items on the screen with the mouse by dragging and dropping. The program automatically saves the input when the screen changes. Feedback from the computer takes the form of either pure text or graphical presentations.

**Figure 2:**
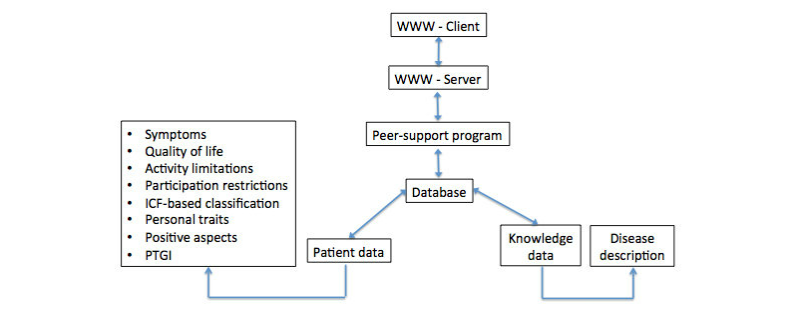
System architecture and data description of the peer-support program. (Note: Questionnaire data is stored in the person-data database and disease descriptors are stored in the knowledge database. The table in the left shows the questionnaires used. The disease descriptors on the right side include the inference engine.).

The peer-support program has been written in PHP-language. It uses a MySQL-database that stores the person data and knowledge data required for verifying the person’s diagnosis. The program generates interactive www-pages with the help of the Apache www-server. Finally, the server sends the pages to the person’s www-client program. The input from the individual is directed to the peer-support program using xhtml forms. The high-level system architecture is presented in the middle of Figure 2.

### Entry Criteria

#### Entry Criteria Based On the American Academy of Otolaryngology-Head and Neck Surgery (AAO-HNS) Criteria

Candidates belonging to the FMF were classified based on the AAO-HNS criteria for Ménière’s disease. The criteria show either definite or probable Ménière’s disease (Table 1). Thus, if the criteria were probable of Ménière’s disease, 35 people with possible Ménière’s disease would be excluded.

**Table 1:** Exclusion and inclusion criteria and their relationship between AAO-HNS-based symptom classifications.

Criteria	Limit	Accepted	Rejected	Missing data
AAO-HNS criteria	Probable or definite	694	35	11
ONE-program	Diagnostic criteria	706	19	15
EQ-5D	VAS score <95%	709	26	5
Disease-specific impact	Impact >0	692	28	20

#### Entry Criteria Based On the ONE-Program Criteria

**Figure 3:**
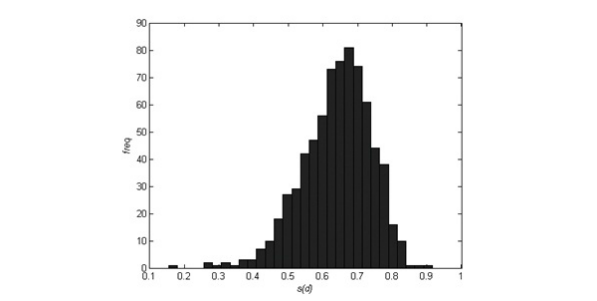
Histogram of program criteria values S(d).

The algorithm of the inference engine compares the diagnostic criteria and symptoms between 14 different conditions and provides fitness scores for each condition. The program suggests the condition with the highest score as the principal diagnosis and provides as diagnostic alternatives the second and third possibilities if their scores exceed a probability of 0.5. Out of the 740 cases, the program accepted 706 as Ménière’s disease and 19 cases were rejected. In all cases, however, Ménière’s disease was the most likely diagnosis. The rejection was done on the basis of the score *S(d)* mentioned above. If the score *S(d)* was more than 0.43, the person was allowed to attend the program. In Table 2, variables are presented where the differences in mean values were statistically significant in variance analysis at a risk level of *P*<.001 between the groups, where *S(d)* was either less than or over 0.43. A histogram of *S(d)* scores is presented in Figure 3.

**Table 2:** Statistically different variables in candidates excluded and included in the computerized peer-support program. (Note: The values belong to the interval [0 x], where 0 means no problem and x means significant problems. Depending on the question, x can have values from 3 to 5.)

Variable	Mean *S(d)*<0.43, excluded	Mean *S(d)*>0.43, included
Age, y	72	62
Frequency of vertigo	0.57	1.96
Duration of vertigo attacks	0.90	2.85
Severity of vertigo	1.0	3.46
Vomiting	0.43	2.22
Balance	2.22	1.31
Severity of unsteadiness	1.67	1.04
Moving ability	1.10	0.48
Raising up from a chair	0.90	0.46

#### Entry Criteria Based On Quality of Life and Impact of the Disease

In order to enter the peer-support program, the condition should have an impact on the person. In the quality of life measurement, if the visual analogue scale (VAS) score is 95% or less, the person can access the peer-support program. With VAS scores greater than 95%, the person would not benefit from the intervention but will still be able to access the information package. With the data from the pilot group, all candidates had Ménière’s disease but their *S(d)* scores varied between 0.15 and 0.92 while the maximum value was 1. The higher the score, the better the match to Ménière’s disease. However, low *S(d)* values might indicate that the person can benefit more from another form of rehabilitation. For instance, a personally tailored and conducted physical training regime could be an appropriate choice. Candidates with an *S(d)* value that’s less than 0.43 are older and their vertigo characteristics are less severe than those with an *S(d)* that’s more than 0.43. The reasoning for a certain threshold is difficult, but in this work we determined the threshold value statistically. By using two standard deviations we cut the left “tail” of the low-end distribution (Figure 3). This excludes 2.6% of candidates from the peer-support program. On the basis of Table 2, these candidates are also seen to have less severe symptoms than the others.

## Results

### Overview

Based on these criteria, 26 of the 740 cases were excluded from the program. The impact of Ménière’s disease was evaluated by the question: “How much does your Ménière’s disease affect your life?” With 28 participants, there was no impact and they were thus rejected from the program.

On the basis of AAO-HNS and ONE-program criterion, 12 candidates were the same. However, when comparing the rejected cases based on ONE-program and EQ-5D, only 1 person was same in both rejection criteria. ONE-program and disease-specific impact shared 18 candidates with matching rejection criteria.

All of the rejected candidates were provided with access to person-to-person peer support and given a provider’s name and contact details. Table 1 summarizes the entry criteria and number of subjects. When a person is accepted into the program, the human gatekeeper provides access codes by email and the person can start the intervention. The age, vertigo, and balance problem variables of the candidates not accepted to the program and of those who were are shown in Table 2.

### Computer Interface in the Questionnaires

In the program, the person is asked to select the severity of his or her activity limitations and participation restrictions. Subsequently they are asked to rate these in such a way that the most severe comes first. The peer-support program then deals with this individual rating of their most problematic limitations and restrictions.

### Positive Aspects

An important aspect of the program is to introduce a positive attitude toward disablement. Positive aspects will improve the participant’s quality of life [17] and may help the person to move their attention to coping strategies [39]. The peer-support program contains 26 examples of possible positive aspects of the disease, which are based on data collected from 560 people belonging to the FMF who were asked to score each positive aspect on a 4-point scale and then rate them in such way that the most important positive aspect came first.

### Feedback on Symptoms When Compared to Average Ménière’s Disease

**Figure 4:**
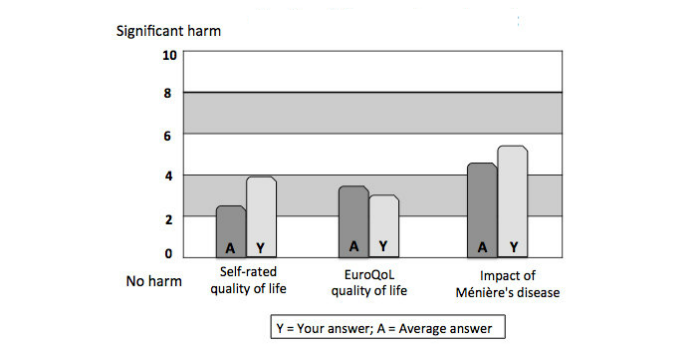
The general health-related quality of life and the disease-specific impact of Ménière's disease compared with 50 matched cases.

To demonstrate the severity of the person’s symptoms compared to other cases, the program provides information on the individual’s severity of the symptoms in comparison to the average of replies from all participants. These are presented in a similar manner as the quality of life aspects depicted in Figure 4.

### Profiling the Person Based on Their Symptoms

The individual profiling based on symptoms was carried out with algorithms using the nearest neighbor classification. This looks for the 50 most similar cases based on symptoms and complaints. The similarity measure is the Euclidean distance between the person profile vectors. In this algorithm, the distance between identical symptom profiles is zero. In order to prevent any component of the profile vector from dominating the distance calculation, all components are divided by their respective maximum values. In order to determine the role of personal trait and attitude, further profiling was used. Both of these individual profiles were related to the General Health Related Quality of Life generic health measure criteria EQ-5D.

In order to have a reasonable number of profiles, participants were profiled on the basis of cardinal symptoms. A person profile is presented in a vector x=[*x*_1_,…,*x*_10_]^T^ where the individual components from the beginning to the end were age, severity of vertigo, intensity of vertigo, frequency of vertigo, severity of walking difficulties, difficulty to attend a conversation, severity of hearing loss, severity of tinnitus, severity of hyperacusis, and severity of the feeling of pressure.

The person profile informs the impact of the condition when related with their personal performance. This information provides feedback on the personal reaction toward the disorder. It informs how personal attitudes, moods, and personality traits modify the suffering from the disorder, and how the person relates him/herself to the context of the illness. Any difference observed will aid the person to realize the difference between problems with their complaints and their consequences in life. In Figure 4, the symptom profile of a patient and the reference group is similar, but the quality of life varies.

The attitude toward the disease is evaluated by displaying the anxiety and SOC scores, and by comparing the scores from 50 reference cases in a converted Likert scale. According to Stephens et al [40], anxiety was an important factor in explaining a reduced quality of life. This data can be used in the program to analyze the need of attitude change and to instruct the person to develop a positive attitude.

## Discussion

### Principal Findings

The current paper presents information about the development of an intelligent computer-based peer-support system that is capable of assessing Ménière’s disease and profiling the impact of the condition. Disease profiling is an important step in management planning and this information is useful in tailoring rehabilitation to meet the needs of a specific individual. Apart from 1 tinnitus program [15], to our knowledge there are no other peer-support programs that deal with inner-ear functions and related difficulties. The novelty of a computerized peer-support program for Ménière’s disease is that it uses artificial intelligence to assess the person’s complaints, profile the disease, and define the individual impact of the condition. The program is constructed to allow the person to focus on the individual impairments and compares these with 50 cases with similar complaint profiles. In the further development of the program, the participants will work interactively with their restrictions and empower themselves to adjust to the restrictions. The program is based on real data retrieved from people with Ménière’s disease. During development of the program, several disease-specific aspects have been documented [39,41-44]. The validation of the program will be presented in our upcoming publications.

### Comparison With Prior and Other Work

#### Focus on Anxiety and Personal Trait

Several investigations have evaluated the psychological aspects of Ménière’s disease [40,45-48]. In general, the authors have focused on mood, anxiety, and personality traits. Söderman et al have indicated that personality traits measured with sense of coherence scale correlated with disability in Ménière’s disease [45]. Hägnebo et al evaluated the psychological correlates of vertigo attacks and studied 514 subjects diagnosed with the condition [47]. Principal component analyses showed that the somatic sensation scale could be divided into 2 subscales: (1) dizziness, vertigo, and anxiety, and (2) sensations in the ear. The psychological state scale showed an energy/awareness factor and a negative emotional state factor. The situation characteristics scale showed 2 factors: (1) environmental disturbances and (2) stressful conditions. Thus they considered fatigue as a psychological component of the disability, whereas anxiety was seen as a somatic component. Vitality is a key component in several indices measuring quality of life (eg, 15D, SF-36), as well as in the perception of “wellness” [49].

Levo et al reported that among subjects with Meniere’s disease, about 70% of the subjects felt fatigued and in 30% this fatigue was moderate to strong [50]. Fatigue was associated with a reduction in general health-related quality of life and also associated with vertigo attacks, balance problems, and hearing loss. The authors concluded that among consequential factors, mobility and anxiety were the strongest consequences of reduced vitality. Among predisposing factors, a sense of coherence and attitude were significant predictors. Further analysis based on participation restrictions indicated that isolation is a severe consequence of a reduction of vitality. Based on the observations of Levo et al, we made an effort in this peer-support program to bring both fatigue and anxiety into the participants’ awareness and by working with the computer program to interactively guide participants to more positively change their attitude to regain social activity [50]. In chronic persistent diseases such as Ménière’s disease, it has been suggested that the evaluation and appraisal of stressors and their subsequent management will improve coping with the disease [51]. In chronic Ménière’s disease, reduced vitality is a common complaint and, especially in its severe form, it should be recognized and included in any therapeutic procedures that are undertaken.

Ménière’s disease is a chronic, progressive disorder that is defined and diagnosed based on the patient’s clinical symptoms. However, previous studies have shown no consistent temporal pattern between the perceived levels of stress and any of the core symptoms of the disease [52]. In Ménière’s disease, patients without significant vertigo spells or incidents of psychological signs such as depression and anxiety associated with the dizziness handicap are comparable to that of a control group. In addition, individual differences emerge and the time resolution for analyzing the association of stress and the psychological aspects associated with the Ménière’s disease may require relatively long-term observation. However, the reciprocal connection between psychological factors and complaint behavior in Ménière’s disease is difficult to resolve [46,53], as among people with Ménière’s disease, personality trait and anxiety were interrelated [50]. Van Cruissen et al indicated that the psychological profile of Ménière’s disease patients seems comparable to patients with other chronic conditions [53]. In their study, Kirby and Yardley indicated that their Ménière’s disease group reported more depression and “health anxiety” than the healthy control group, and that “health anxiety” was associated with anxiety and depression [46]. After controlling for the severity of the symptoms, anxiety was associated with several illness perception subscales, including emotional representations, consequences, psychological causes, and the tolerance of uncertainty. Contrary to this, however, Brantberg and Baloh considered fatigue to be a trigger for Ménière’s disease [54]. In line with Kirby and Yardley [46] and Levo et al [50], in the computerized peer-support program we consider that the reduction in vitality is a consequence of the condition (in this case vestibular dysfunction) rather than a causative factor. Personality trait has been also been regarded as a modifying factor for the condition [50]. Moreover, the relatively minor role of personality trait in quality of life and disease-specific impact has been previously documented [40,45,46].

An optimistic view of the future results in better adjustment outcomes, since optimists tend to continue with their adaptive coping efforts when confronted by adversity [55]. In Ménière’s disease, we observed that a positive attitude improves coping with the condition and improves quality of life [17,19]. It is of note that the subjects did not identify any wishful thinking (ie, a miracle improvement) or escape-avoidance coping (ie, trying to forget the disease) that have been otherwise regarded as strong predictors for mal-adjustments in neurological disease [51]. A positive attitude has been associated with lower depression, less anxiety [56], and better physical and social adjustment [57,58]. By bringing up positive aspects in the peer-support program, we aim to improve coping. However, before launching the program to a wider market, the validity and user satisfaction of the program must be certified. For validation we are using changes in the quality of life instrument [21] and in the post-traumatic growth inventory [24].

#### Computer-Based Peer-Support Programs

Several computerized peer-support programs have been recently launched on the Internet. For instance, programs on diabetes care [59], coping with bulimia [60], bipolar mood disorders, anxiety (eg, Beat the Blues, Cope) [61], phobias and panic (eg, FearFighter) [62], obsessive-compulsive disorder (eg, BT Steps) [61], and tinnitus [15] are now present. 

All of these programs use questionnaires and provide instructions to stimulate behavioral changes in order to cope better with the featured condition. The effectiveness of Beat the Blues, Cope, FearFighter, and BT Steps have been considered from a cost/benefit point of view [61]. Computerized treatment can yield savings and provide benefits for patients that are comparable to the therapy provided by physicians [15,61]. The effect of the tinnitus peer-support program for the person was found to be as effective as that of a personal support program [62].

By definition, the expert system is a set of programs that manipulates encoded best knowledge to solve a problem in a specialized domain that normally requires human expertise [35,63]. The program is adaptive and learns during use (ie, a new person’s data updates the database). The solutions to problems will converge to the best possible solutions available during the course of the program. If information is not appropriate for the condition, it will not be included in the alternative diagnostic choice.

A symptom-based classification method is recommended by AAO-HNS [64] to make the diagnosis, and this has been used in the present study. Indeed, in a taxonomic investigation of patients with vertigo, after the exclusion of neurological and middle-ear conditions, head trauma, and ototoxicity, Hinchcliffe has found that those with “classical” Ménière’s disease (ie, those meeting the “probable” definition based on the AAO-HNS definition) fell into a single nosological entity with all other cases of vertigo [65]. He later argued that Ménière’s disease included “formes frustes,” where the triad of symptoms is not complete [65]. The AAO-HNS has proposed the currently used classification. It defines “possible Ménière’s disease,” “probable Ménière’s disease,” and “definite Ménière’s disease.” “Certain Ménière’s disease” is diagnosed by the symptom entity and histological verification of endolymphatic hydrops in the inner ear. To define the condition clinically, however, the existing AAO-HNS classification is unhelpful [65]. In a recent study, Pyykkö et al used MRI to diagnose endolymphatic hydrops and found that all patients with definite Ménière’s disease had endolymphatic hydrops [66]. Patients with probable Ménière’s disease had endolymphatic hydrops in 95% of cases. Thus, the current criteria for inclusion of people with definite and probable Ménière’s disease into the program seems to be quite accurate, although some cases may be erroneously classified. In a previous study we compared the accuracy of the inference engine and different vertiginous disease diagnoses made by human experts [67]. With the same set of data, the current inference engine correctly diagnosed 65% of the cases, while younger physicians succeeded in 54% and the experienced physician in 65% of the cases [67].

### Program Design

#### User Interface

The selected user interface solutions follow the ideas presented in the work of Hawthorn [68]. Hawthorn stated that most difficulties in the use of a computer originate from a degradation of vision, cognitive ability, and motor control. In our program, the automatic data saving removes the burden from short-term memory, and stationary screens are easy to follow and minimize the need for scrolling, which helps users who may have problems with their motor control.

One major problem is how to construct simple and clear sentences for the questions. In addition, a certain amount of guidance is needed in problematic situations. The program contains help topics and best practices drawn from other participants. If a mouse remains stationary on the questionnaires, a short tooltip shows additional help. This help is available either as a short text or as an example form that shows what the user should do next. The help information docks on the particular page and does not branch the program flow. This prevents the user getting lost in the program. The task in question is always visible on the screen.

#### Individual Learning

The questionnaires in the program also contain open-ended questions and fields for the person’s replies. These replies are stored in the database. The open-ended questions are ultimately used in providing useful self-help items and coping strategies [39,42]. After completing each questionnaire, the person is informed how he/she compares with other persons. The purpose of feedback is 3-fold: (1) it tells the user where he/she stands with their problems when compared to other participants [18]; (2) it enables the progress of the program [35]; and (3) it reinforces learning and motivation [69]. In some self-help programs [15,62], the commitment of users appears to present a problem.

A usual method to reinforce learning is to use forced-choice tasks. By focusing on 3 major problems and ranking them, the program helps the person to focus and identify the major impacts of the disease on his/her life [41,44]. In this decision-making process, the person ranks the impacts and tries to maximize the beneficial outcomes. In the program, these actions are continuously revised by the application of strategies leading to improvement [70]. This type of reinforcement learning leads parsimoniously to improvement as indicated by behavioral and neurophysiological studies in humans [69]. In addition to problems classified based on ICF, the program also uses positive experiences to reinforce attitude and personal trait [17,71]. Neurophysiological theories indicate that by reinforcing positive experiences, new synapses are established and the synaptic efficacy is improved. This has a stabilizing effect on the organism, either via improved neural transmission or via targeted synaptic triads [72]. Thus, the use of positive aspects will break down negative reinforcement and allow the nervous system to recover and learn new rules. In this manner, positive aspects work as rewards and allow internal evaluations of covert motor actions without defining them as behaviors [73].

Most often, expert teams build these programs without establishing any solid data collection from individuals. Our computerized peer-support program for Ménière’s disease offers a difference, in that it uses an inference engine based on pattern recognition to reveal the accuracy of the diagnosis. It also profiles individual cases with a nearest neighbor classification algorithm and provides peer-support based on real-person oriented data.

### Conclusions

We have developed a computerized peer-support program that can verify and assess the diagnosis of Ménière’s disease by using a pattern recognition method. The program can function even with partial data. The database is continuously utilized and updated by the program, and uses a knowledge engine to offer solutions to a person’s problems. Such a system can be very helpful when assessing and diagnosing chronic conditions that are diagnosed based on symptoms, rather than detailed clinical investigations.

## Abbreviations

FMF: Finnish Ménière’s Federation
HDHS: hearing disability and handicap scale
HMS: hearing measurement scale
ICF: International Classification of Functioning, Disability, and Health
ITI: international tinnitus inventory
PTGI-SF: short form of the post-traumatic growth inventory
SF-36: Short Form Health Survey
SOC: sense of coherence
VAS: visual analogue scale
